# Evaluation of nutritional status in pediatric intensive care unit patients: the results of a multicenter, prospective study in Turkey

**DOI:** 10.3389/fped.2023.1179721

**Published:** 2023-08-03

**Authors:** Merve Misirlioglu, Dincer Yildizdas, Faruk Ekinci, Ozden Ozgur Horoz, Gokhan Tumgor, Ahmet Yontem, Mehmet Nur Talay, Murat Kangin, Erennur Tufan, Selman Kesici, Nazik Yener, Hatice Elif Kinik Kaya, Merve Havan, Ali Tunc, Nihal Akçay, Esra Sevketoglu, Fatih Durak, Aysenur Ozel Dogruoz, Serhan Ozcan, Oktay Perk, Muhterem Duyu, Merve Boyraz, Mutlu Uysal Yazici, Zeynelabidin Ozturk, Mehmet Çeleğen, Aysegul Bukulmez, Ebru Kacmaz, Ener Cagri Dinleyici, Oguz Dursun, Alper Koker, Suleyman Bayraktar, Mey Talip Petmezci, Aygul Nabaliyeva, Hasan Agin, Pinar Hepduman, Emine Akkuzu, Tanil Kendirli, Hasan Ozen, Sevgi Topal, Çağlar Ödek, Murat Ozkale, Yasemin Ozkale, Gürkan Atay, Seher Erdoğan, Capan Konca, Guler Yapici, Gazi Arslan, Tolga Besci, Resul Yilmaz, Meltem Gumus, Arzu Oto, Tahir Dalkiran, Mehmet Mercan, Yasemin Çoban, Sevcan Ipek, Sukru Gungor, Ali Ertug Arslankoylu, Mehmet Alakaya, Ferhat Sari, Aylin Yucel, Abdullah Yazar

**Affiliations:** ^1^ Department of Pediatric Intensive Care, Faculty of Medicine, Mersin University, Mersin, Türkiye; ^2^ Department of Pediatric Intensive Care, Faculty of Medicine, Cukurova University, Adana, Türkiye; ^3^ Department of Pediatric Gastroenterology, Hepatology and Nutrition, Faculty of Medicine, Cukurova University, Adana, Türkiye; ^4^ Department of Pediatrics, Health Sciences University, Gazi Yasargil Training and Research Hospital, Diyarbakir, Türkiye; ^5^ Department of Pediatric Intensive Care, Faculty of Medicine, Medipol University, Istanbul, Türkiye; ^6^ Department of Pediatric Intensive Care, Faculty of Medicine, Hacettepe University, Ankara, Türkiye; ^7^ Department of Pediatric Intensive Care, Faculty of Medicine, Ondokuz Mayıs University, Samsun, Türkiye; ^8^ Department of Pediatric Intensive Care, Mersin City Training and Research Hospital, Mersin, Türkiye; ^9^ Department of Pediatrics, Mersin City Training and Research Hospital, Mersin, Türkiye; ^10^ Department of Pediatric Intensive Care, University of Health Sciences Bakirkoy, Dr. Sadi Konuk Training and Research Hospital, İstanbul, Türkiye; ^11^ Department of Pediatric Intensive Care, İzmir Health Sciences University, Tepecik Training and Research Hospital, İzmir, Türkiye; ^12^ Department of Pediatric Intensive Care, Ankara City Hospital, Ankara, Türkiye; ^13^ Department of Pediatric Intensive Care, Medeniyet University, Prof. Dr. Suleyman Yalcin City Hospital, Istanbul, Türkiye; ^14^ Department of Pediatric Intensive Care, Health Sciences University Ankara, Dr. Sami Ulus Obstetrics Child Health and Diseases Training and Research Hospital, Ankara, Türkiye; ^15^ Department of Pediatric Intensive Care, Faculty of Medicine, Afyonkarahisar Health Sciences University, Afyonkarahisar, Türkiye; ^16^ Department of Pediatric Gastroenterology, Faculty of Medicine, Hepatology and Nutrition, Afyonkarahisar Health Sciences University, Afyonkarahisar, Türkiye; ^17^ Department of Pediatric Intensive Care, Faculty of Medicine, Eskisehir Osmangazi University, Eskisehir, Türkiye; ^18^ Department of Pediatric Intensive Care, Faculty of Medicine, Akdeniz University, Antalya, Türkiye; ^19^ Department of Pediatric Intensive Care, Sultangazi Haseki Training and Research Hospital, Istanbul, Türkiye; ^20^ Department of Pediatric Intensive Care, Prof. Dr. Cemil Tascıoglu City Hospital, Istanbul, Türkiye; ^21^ Department of Pediatric Intensive Care, University of Health Sciences Izmir, Dr. Behcet Uz Child Diseases and Surgery Training and Research Hospital, Izmir, Türkiye; ^22^ Department of Pediatric Intensive Care, Isparta City Hospital, Isparta, Türkiye; ^23^ Department of Pediatric Intensive Care, Faculty of Medicine, Ankara University, Ankara, Türkiye; ^24^ Department of Pediatric Intensive Care, Erzurum Regional Training and Research Hospital, Erzurum Türkiye; ^25^ Department of Pediatric Intensive Care, Faculty of Medicine, Uludag University, Bursa, Türkiye; ^26^ Department of Pediatric Intensive Care, Faculty of Medicine, Baskent University, Adana Dr Turgut Noyan Teaching and Medical Research Center, Adana, Türkiye; ^27^ Department of Pediatric Intensive Care, Umraniye Training and Research Hospital, Istanbul, Türkiye; ^28^ Department of Pediatric Intensive Care, Adiyaman Training and Research Hospital, Adiyaman, Türkiye; ^29^ Department of Pediatric Intensive Care, Faculty of Medicine, Dokuz Eylul University, Izmir, Türkiye; ^30^ Department of Pediatric Intensive Care, Faculty of Medicine, Selcuk University, Konya, Türkiye; ^31^ Department of Pediatric Gastroenterology, Hepatology and Nutrition, Faculty of Medicine, Selcuk University, Konya, Türkiye; ^32^ Department of Pediatric Intensive Care, University of Health Sciences Bursa High Specialization Hospital, Bursa, Türkiye; ^33^ Department of Pediatric Intensive Care, Necip Fazil City Hospital, Kahramanmaras, Türkiye; ^34^ Department of Pediatrics, Necip Fazil City Hospital, Kahramanmaras, Türkiye; ^35^ Department of Pediatric Intensive Care, Faculty of Medicine, Mugla University, Mugla, Türkiye; ^36^ Department of Pediatric Intensive Care, Faculty of Medicine, Sutcu Imam University, Kahramanmaras, Türkiye; ^37^ Department of Pediatric Gastroenterology, Hepatology and Nutrition, Faculty of Medicine, Sutcu Imam University, Kahramanmaras, Türkiye; ^38^ Department of Pediatric Intensive Care, Faculty of Medicine, Hatay Mustafa Kemal University, Hatay, Türkiye; ^39^ Department of Pediatric Gastroenterology, Hepatology and Nutrition, Faculty of Medicine, Necmettin Erbakan University Meram, Konya, Türkiye; ^40^ Department of Pediatric Intensive Care, Faculty of Medicine, Necmettin Erbakan University Meram, Konya, Türkiye

**Keywords:** calorie intake, malnutrition, nutrition, pediatric intensive care unit, protein intake

## Abstract

**Introduction:**

Malnutrition is defined as a pathological condition arising from deficient or imbalanced intake of nutritional elements. Factors such as increasing metabolic demands during the disease course in the hospitalized patients and inadequate calorie intake increase the risk of malnutrition. The aim of the present study is to evaluate nutritional status of patients admitted to pediatric intensive care units (PICU) in Turkey, examine the effect of nutrition on the treatment process and draw attention to the need for regulating nutritional support of patients while continuing existing therapies.

**Material and Method:**

In this prospective multicenter study, the data was collected over a period of one month from PICUs participating in the PICU Nutrition Study Group in Turkey. Anthropometric data of the patients, calorie intake, 90-day mortality, need for mechanical ventilation, length of hospital stay and length of stay in intensive care unit were recorded and the relationship between these parameters was examined.

**Results:**

Of the 614 patients included in the study, malnutrition was detected in 45.4% of the patients. Enteral feeding was initiated in 40.6% (*n* = 249) of the patients at day one upon admission to the intensive care unit. In the first 48 h, 86.82% (*n* = 533) of the patients achieved the target calorie intake, and 81.65% (*n* = 307) of the 376 patients remaining in the intensive care unit achieved the target calorie intake at the end of one week. The risk of mortality decreased with increasing upper mid-arm circumference and triceps skin fold thickness Z-score (OR = 0.871/0.894; *p* = 0.027/0.024). The risk of mortality was 2.723 times higher in patients who did not achieve the target calorie intake at first 48 h (*p* = 0.006) and the risk was 3.829 times higher in patients who did not achieve the target calorie intake at the end of one week (*p* = 0.001). The risk of mortality decreased with increasing triceps skin fold thickness Z-score (OR = 0.894; *p* = 0.024).

**Conclusion:**

Timely and appropriate nutritional support in critically ill patients favorably affects the clinical course. The results of the present study suggest that mortality rate is higher in patients who fail to achieve the target calorie intake at first 48 h and day seven of admission to the intensive care unit. The risk of mortality decreases with increasing triceps skin fold thickness Z-score.

## Introduction

Malnutrition is frequent in the hospitalized pediatric patients. Critically ill children are even at higher risk of developing malnutrition and the majority have malnutrition at the time of hospitalization or develop acute and/or chronic malnutrition during the disease course (1–3). Malnutrition in the pediatric intensive care unit (PICU) patients is associated with increased mortality and morbidity such as predisposition to infections, impairment in gastrointestinal functions, prolonged hospitalization and prolonged mechanical ventilation (4).

Nutritional history of the patients must be gathered, appropriate energy requirements must be calculated and anthropometric data must be obtained to provide appropriate nutritional support. Nutrition guidelines recommend all children admitted to the intensive care unit undergo screening for nutritional status, monitorization of children for possible risk of malnutrition and specify a support plan in the presence of malnutrition. Metabolic demands must be determined by calculating energy consumption, feeding protocols must be followed by creating specialized feeding support teams and early enteral feeding must be provided as long as contraindications do not exist (5). The aim of the present study is to analyze anthropometric data, nutritional status, enteral and parenteral feeding support, and calorie and protein intake of critically ill pediatric patients in Turkey and evaluate the relationship between nutritional support and clinical outcomes.

## Material and method

### Study population and design

In the scope of this multicenter, prospective study involving 33 pediatric intensive care units in 18 provinces of Turkey, all patients recently admitted to the intensive care unit during a one-month period were enrolled. Among 1,007 patients admitted to the PICUs during a one-month period, a total of 381 patients who were younger than one month or older than 18 years, who had a corrected age of less than one month, who stayed in the intensive care unit less than 48 h or who had recurrent admissions were excluded from the study. If the patient had recurrent admissions to the PICU during the specified study period, only the first admission was included in the study. Of the remaining 626 patients, 12 were excluded because they continued staying in the intensive care unit at the end of the three-month study period, and the data of 614 patients were included in the final analysis.

The study was granted approval by Cukurova University Faculty of Medicine Non-Interventional Clinical Trials Ethics Committee (Decision No: 05.03.2021/109-3). The approval of the ethics committee was disseminated to the participating centers and the approval of the individual hospitals and the voluntary informed consent of the parents of all participating patients were obtained.

### Data collection

The study forms were delivered to the centers participating in the Nutrition Study Group that was established within the body of the Society of Pediatric Emergency and Intensive Care Medicine, and the data was recorded on the forms by two pediatric intensive care unit specialists in each center responsible for the relevant center.

Demographic characteristics and evaluation data of the critically ill children were recorded on these forms. The variables included patient's name and last name, age, gender, diagnosis category, source of admission (directly from home, general ward from the same hospital, pediatric emergency room, transfer from another intensive care unit), length of stay in the pediatric intensive care unit, length of hospital stay, respiratory support, duration of mechanical ventilation and in-hospital mortality. The reason for admission was divided into three groups as medical, emergency surgery and elective surgery. The diagnoses on admission were categorized according to the systems. Disease severity, mortality scores including the Pediatric Index of Mortality (PIM 2) (6) and the Pediatric Risk of Mortality (PRISM) III (7) were calculated upon admission; the highest Vasoactive inotropic score (VIS) (8) measured during the follow-up was recorded.

### Nutritional Status

Anthropometric data of the study patients was recorded on the day of admission to the PICU and the day of discharge. Height, body weight, triceps skin fold thickness, mid-upper arm circumference (MUAC) of children were measured. Mid-upper arm circumference was measured using a nonelastic measuring tape, and the triceps skin fold thickness was measured using a device called caliper. The measurements were performed by a calibrated devices using standard techniques and the reminders of standard techniques were delivered to the participating centers before starting the study. The data collected was used to calculate height-for-age, weight-for-age, weight-for-height percentiles and body mass index Z-scores to evaluate nutritional status of the patients. Using the body weight and height measurements, body mass index (BMI) was calculated by dividing the weight (kg) by the square height (m) in meters. The standard deviation scores (Z-scores) for height, body weight, head circumference and body mass index were calculated using the CHILD METRICS software to standardize the study data (9, 10). The degree of malnutrition is assessed clinically using various anthropometric measurements. The assessment methods are based upon the assumption that during periods of nutritional deprivation, weight deficits occur initially, followed by faltering length or height and, finally, by lagging head circumference growth. The severity of wasting or stunting is defined by comparing a child's weight and height measurements with those of a population reference standard. The patient's nutritional status is evaluated by calculating the body mass index (BMI) Z-score (<2 years, weight for height Z-score) by measuring the body weight and height of the patient during hospitalization in the pediatric intensive care unit. If the patient's height is unknown, the patient's nutritional status is evaluated by calculating the weight Z-score for age. There are different classifications for assessing the degree of malnutrition; the GOMEZ classification uses weight for age, the Waterlow classification uses height for age, and the WHO classification uses weight for height. In children with ascites and edema, anthropometric measurements that are not affected by these, such as middle-upper arm circumference and triceps skin fold thickness, should be preferred. For infant under 6 months of age, weight for age Z score (WAZ)<−3 SD, weight for length Z score(WLZ)<−3 SD criteria may be used to define severe acute malnutrition. For children 6–59 months age group, diagnostic criteria are; severe acute malnutrition: MUAC < 115 mm, or WLZ < −3 SD; moderate acute malnutrition: WLZ −2 to −3 SD, or MUAC 115 to 124 mm; chronic malnutrition: length Z score < −2SD. For children over five years of age and adolescents, the WHO recommends the use of body mass index-for-age Z-scores to screen for malnutrition. Alternatively, MUAC-for-age Z-score charts for children between 5 and 19 years have been developed (11, 12).

Nutritional status of the patients, number of days without feeding, the first day of starting feeding, reasons for enteral feeding, the amount of energy and protein intake in the first 48 h, first one week and on discharge, and whether or not the desired target calorie and protein intake has been reached were recorded on the data collection forms. The target calorie intake was calculated according to the body weight using the Schofield equation (Schofield-W) (13).

### Measurement techniques

Body weight measurement is frequently used as an indication of nutritional status. During the measurement, all clothing was removed in small children and the weight of older children was measured in underwear. The measurements were made before feeding when the child was fasting. Body weight was recorded in kilograms (kg). Height measurements were obtained with the child placed in supine position to standardize the measurements due to difficulties in the follow-up of critically ill patients who are unable to remain standing most of the time. While the child was placed in supine position during height measurement at the level of soles, one operator held the head of the child with the vertex in close contact with the stable edge of the bed and the second operator stabilized the child's legs with the knees extended. Particular attention was paid to the feet being naked during the measurement with the hips and shoulder blades rested against the surface and the heels kept together. Height was recorded in centimeters (cm) (14).

During the measurement of the mid-upper arm circumference, a mark was placed on the acromion while the child was seated in upright position as much as possible with the elbow flexed 90 degrees and the palms facing the floor. The distance between the olecranon and acromion was determined, then the arm was released and the measurement was made in millimeters (mm) without making pressure on the arm with the tape placed perpendicular to the arm. The Harpenden skin fold caliper, also called briefly the caliper, was used to measure triceps skin fold thickness. The left elbow was flexed 90 degrees and the midpoint between the acromion and the olecranon was marked. The arm was then released and the skin fold thickness was measured in millimeters (mm) from the marked point using the caliper while grasping the skin fold with the index finger and thumb of the left hand. It was ensured that the caliper has grasped completely the skin and subcutaneous tissues and the movement of the caliper and the skin fold was avoided during the measurement (14). Standard deviation scores (Z-scores) were calculated using the PediTools software to standardize the mid-upper arm circumference and triceps skin fold thickness measurements (15).

### Statistical analysis

The data was analyzed using IBM SPSS version 23 software. The Kolmogorov–Smirnov test and Shapiro-Wilk test were used to evaluate the fitness to normal distribution. The Wilcoxon test was used in the analysis of variables without normal distribution over time. Pearson's chi-square test was used to compare categorical variables and a Z-test with Bonferroni correction was used in the comparison of multiple proportions. The Mann-Whitney *U* test was used to compare paired groups without normal distribution. The Kruskal Wallis H test was used to compare three or more groups without normal distribution and the Dunn's test was used in multiple comparisons. The Spearman's correlation coefficient (rho) was used to examine the relationship between variables without normal distribution. The factors affecting the achievement of target calorie intake at the end of 48 h and at day 7 were examined in univariate models using binary logistic regression analysis. Linear regression analysis was used to examine the independent variables affecting the day of death, length of stay in the PICU, length of hospital stay and duration of mechanical ventilation. Binary logistic regression analysis was used to examine the risk factors affecting mortality and nosocomial infections. The quantitative variables were investigated using visual (histograms and probability plots) and analytical methods (Kolmogorov Smirnov test) to determine whether or not they are normally distributed. At the end of the analysis, quantitative data was expressed as mean ± standard deviation if normally distributed, and median (minimum-maximum) if not normally distributed*.* Categorical data was expressed as frequency and percentage. A *p*-value of less than 0.05 was considered statistically significant.

## Results

In this countrywide prospective study that was conducted in Turkey, the data of 614 patients, who were admitted to the intensive care unit during a one-month study period and who met the study inclusion criteria, was examined. Of these patients, 54.7% (*n* = 336) were male and the mean age was 80.17 ± 68.35 months. The reason for admission to the intensive care unit was medical causes in 76.2% (*n* = 468) and emergency surgery in 12.2% (*n* = 75) of the patients. Of the patients, 62.7% (*n* = 385) had an underlying chronic condition. Descriptive statistics according to the reason for admission, source of admission, diagnoses and the underlying conditions are presented in Table 1.

**Table 1:** Demographic characteristics of the patients.

	Frequency (*n*)	Percentage (%)
Gender		
Female	278	45.3
Male	336	54.7
Reason for PICU Admission		
Medical	468	76.2
Emergency Surgery	75	12.2
Elective Surgery	71	11.6
The Route of Admission to PICU		
Admission from the Pediatric Emergency Room	368	59.9
Other Clinics in the Hospital	169	27.5
Transfer from Other Intensive Care Units	63	10.3
Admission from Home	14	2.3
Diagnosis on Admission to PICU		
Postoperative Care	145	23.6
Respiratory Failure	143	23.3
Hemodynamic Unstability/Shock	114	18.6
Intoxication	62	10.1
Status Epilepticus	55	9
Trauma	22	3.6
Diabetic Ketoacidosis	18	2.9
Postarrest Care	13	2.1
Other	42	6.8
Underlying Condition		
None	226	37.3
Neurologic/Neuromuscular Disease	93	15.1
Cardiovascular Disease	69	11.2
Metabolic Disease	47	7.7
Congenital/Genetic Syndrome	38	6.2
Hematologic/Immunologic Disorder	35	5.7
Malignancy	33	5.4
Technology-dependent Child	19	3.1
Gastrointestinal Disease	18	2.9
Respiratory Diseases	13	2.1
Renal/Urological Disease	8	1.3
Transplant Recipient	7	1.1
Prematurity/Neonatal Disorders	5	0.8

PICU, pediatric intensive care unit.

The study found that 45.4% (*n* = 279) of the patients admitted to the pediatric intensive care unit had malnutrition according to weight for age classification. Of the patients, 23.0% (*n* = 141) had chronic malnutrition. The classification of nutritional status according to the duration of malnutrition, height-for-age, weight-for-age, weight-for-height, body mass index (BMI) Z-score, mid-upper arm circumference Z-score and triceps skin fold thickness Z-score is presented in Table 2. The body weight Z-scores upon admission to the PICU were compared with the Z-score on discharge in the groups created according to the BMI Z-score. There was a significant difference between median body weight Z-score upon admission to the PICU and on discharge in severely underweight patients (*p* = 0.019); the differences in the other groups were not statistically significant (*p* > 0.050) (Table 3).

**Table 2:** Classification of the patients according to nutritional status.

	*n* (%)
Weight-for-height	
Severe malnutrition	39 (6.4%)
Moderate malnutrition	53 (8.6%)
Mild malnutrition	97 (15.8%)
Normal	289 (47.1%)
Overweight	61 (9.9%)
Obese	75 (12.2%)
According to BMI Z-score	
Morbidly obese	11 (1.8%)
Overweight	31 (5.0%)
Normal	82 (13.4%)
Underweight	355 (57.8%)
Severely underweight	78 (12.7%)
Weight-for-age	
Normal	335 (54.6%)
Mild malnutrition	124 (20.2%)
Moderate malnutrition	97 (15.8%)
Severe malnutrition	58 (9.4%)
Height-for-age	
Normal	358 (58.3%)
Mild malnutrition	133 (21.7%)
Moderate malnutrition	55 (9.0%)
Severe malnutrition	68 (11.1%)
Duration of Malnutrition	
Normal	285 (46.4%)
Acute-underweight	92 (15.0%)
Acute-Chronic malnutrition	96 (15.6%)
Chronic-Short stature	141 (23.0%)
According to Mid-Upper Arm Circumference Percentile	
Malnutrition (below 5th percentile)	235 (38.3%)
Normal (5th–90th percentile)	338 (55.0%)
Obese (above 90th percentile)	41 (6.7%)
According to Triceps Skinfold Thickness Percentile	
Malnutrition (below 5th percentile)	145 (23.6%)
Normal (5th–90th percentile)	416 (67.8%)
Obese (above 90th percentile)	53 (8.6%)
Total	614 (100%)

BMI, body mass index.

**Table 3:** Comparison of the BMI groups according to body weight index Z-score upon admission to and discharge from intensive care unit.

	Body Weight Z-score on Discharge	Body Weight Z-score on Admission to PICU	*p* *
Morbidly Obese	3.72 (−1.14–5.32)^c^	3.77 (−1.14–5.32)^c^	0.317
Obese	2.28 (−2–5.37)^c^	2.28 (−1.45–5.37)^c^	0.798
Overweight	0.77 (−5.53–3.12)^c^	0.68 (−5.53–2.6)^c^	0.946
Normal	−0.66 (−7.82–6.2)^b^	−0.64 (−7.66–1.94)^b^	0.056
Underweight	−2.28 (−7.6– −0.19)^a^	−2.21 (−5.44– −0.63)^a^	0.552
Severely underweight	−3.83 (−11–0.61)^a^	−3.83 (−11.87–0.67)^a^	0.019
*p* **	<0.001	<0.001	

BMI, body mass index.

* Wilcoxon test.

** Kruskall-Wallis H test, median (minimum—maximum).

^a–c^ There is no difference between the groups denoted by the same letter.

Enteral feeding was initiated in 40.6% (*n* = 249) of the patients at day one upon admission to the intensive care unit, and 91.5% (*n* = 562) received enteral feeding at the end of one week. The rate of parenteral nutrition was 6.5% (*n* = 40) at day one upon admission to the intensive care unit and 8% (*n* = 30)at the end of one week. The barriers to the delivery of enteral nutrition was intubation/extubation procedures in 36.5% (*n* = 135) and intolerance to enteral nutrition in 30% (*n* = 111). The initiation of enteral nutrition within 48 h upon admission to the intensive care unit is regarded as early enteral nutrition that was achieved in 72.3% (*n* = 444) of the study patients. A comparison of nutritional status between patients initiated on early versus late enteral nutrition is presented in Table 4. No statistically significant difference was found in terms of nutritional status and feeding status according to the initiation of early enteral nutrition (*p* > 0.050).

**Table 4:** Comparison of nutritional status and feeding status according to early enteral feeding.

	No early feeding	Early feeding	*p* *
Nutritional Status According to t BMI Z-score			
Morbidly Obese	2 (1.3)	9 (2)	0.172
Obese	12 (7.8)	19 (4.3)	
Overweight	25 (16.2)	54 (12.2)	
Normal	89 (57.8)	255 (57.4)	
Underweight	13 (8.4)	44 (9.9)	
Severely underweight	13 (8.4)	63 (14.2)	
According to Mid-Upper Arm Circumference Percentile			
Malnutrition (below 5th percentile)	54 (35.1)	174 (39.2)	0.623
Normal (5th–90th percentile)	88 (57.1)	241 (54.3)	
Obese (above 90th percentile)	12 (7.8)	29 (6.5)	
According to Triceps Skinfold Thickness Percentile			
Malnutrition (below 5th percentile)	30 (19.5)	113 (25.3)	0.196
Normal (5th–90th percentile)	113 (73.4)	290 (65.4)	
Obese (above 90th percentile)	11 (7.1)	41 (9.3)	

BMI, body mass index.

* Pearson's chi-square test.

The mean calorie intake at 48 h after admission to the intensive care unit was 43.94 ± 32.26 Kcal/kg/day and the mean protein intake was 1.24 ± 0.94 gr/kg/day. The mean calorie intake at the end of one week after admission to the intensive care unit was 57.39 ± 32.97 Kcal/kg/day and the mean protein intake was 1.61 ± 0.93 gr/kg/day. It was found that the target calorie intake was achieved in 86.82% (*n* = 533) of the patients in the first 48 h after admission. Of the remaining 376 patients, 81.65% (*n* = 307) achieved the target calorie intake at the end one week after admission to the intensive care unit (Table 5). The analysis of diagnoses among critically ill patients who did not achieve the target calorie intake at first 48 h and day seven after admission revealed that trauma and respiratory failure were the most common diagnoses. Intolerance to enteral nutrition occurred in 19.9% (*n* = 122) of the patients. These included vomiting (52%, *n* = 64), abdominal distension (46.3%, *n* = 57), electrolyte imbalance (17.9%, *n* = 22), diarrhea (16.3%, *n* = 20), gastrointestinal hemorrhage (11.4%, *n* = 14) and constipation (8.9%, *n* = 11). The factors affecting the achievement of target calorie intake at the end of 48 h and at day 7 were examined in univariate models using binary logistic regression analysis. Patients who did not receive mechanical ventilation in the first 24 h, who did not have nosocomial infections, who did not receive inotropic therapy and who received peroral nutrition than those receiving tube feeding were more likely to achieve the target calorie intake at 48 h after admission. The likelihood of achieving the target calorie intake at 48 h decreased with increasing VIS, PIM2 score, PRISM III score, patient age, duration of mechanical ventilation and length of stay in the intensive care unit and the hospital. Patients who did not undergo mechanical ventilation in the first 24 h, who did not develop feeding complications, who did not undergo plasmapheresis, who did not receive inotropic therapy and those who did not receive tube feeding were more likely to achieve the target calorie intake at day seven. The likelihood of achieving the target calorie intake at day seven decreased with increasing VIS, PIM2 score, PRISM III score, duration of mechanical ventilation and length of stay in the intensive care unit and the hospital (Tables 6, 7).

**Table 5:** Comparison of nutritional status and feeding status according to the achievement of target calorie intake at 48 h and 7 days.

	Target calorie achievement			Target calorie achievement		
	Yes	No	*p*	Yes	No	*p*
According to BMI Z-score						
Morbidly Obese	9 (1.7)	2 (2.5)	0.247	7 (2.3)	0 (0)	0.019
Obese	26 (4.9)	5 (6.2)		10 (3.3)^a^	6 (8.7)^b^	
Overweight	69 (12.9)	13 (16)		38 (12.4)^a^	16 (23.2)^b^	
Normal	304 (57)	51 (63)		170 (55.4)^a^	36 (52.2)^a^	
Underweight	55 (10.3)	2 (2.5)		35 (11.4)^a^	3 (4.3)^a^	
Severely underweight	70 (13.1)	8 (9.9)		47 (15.3)^a^	8 (11.6)^a^	
According to Mid-Upper Arm Circumference Percentile						
Malnutrition (below 5th percentile)	201 (37.7)	34 (42)	0.663	122 (39.7)	31 (44.9)	0.311
Normal (5th–90th percentile)	295 (55.3)	43 (53.1)		169 (55)	32 (46.4)	
Obese (above 90th percentile)	37 (6.9)	4 (4.9)		16 (5.2)	6 (8.7)	
According to Triceps Skinfold Thickness Percentile						
Malnutrition (below 5th percentile)	135 (25.4)^a^	9 (11.1)^b^	0.008	88 (28.8)	13 (18.8)	0.07
Normal (5th–90th percentile)	349 (65.6)^a^	67 (82.7)^b^		198 (64.7)	47 (68.1)	
Obese (above 90th percentile)	48 (9)^a^	5 (6.2)^a^		20 (6.5)	9 (13)	

BMI, body mass index.

* Pearson's chi-square test.

^a,b^ There is no difference between the groups denoted by the same letter.

**Table 6:** Factors affecting reaching the target calorie intake at 48 h of admission.

	Univariate	
	OR (95% CI)	*p*
Gender (Reference: Female)	1.28 (0.802–2.044)	0.301
Source of Admission to the PICU (Reference: Direct Admission from Home)		
Admission from the Pediatric Emergency Room	1.695 (0.457–6.285)	0.430
Other Clinics in the Hospital	2.608 (0.658–10.331)	0.172
Transfer from Other Intensive Care Units	1.289 (0.308–5.402)	0.728
Comorbid disease (Reference: Present)	1.013 (0.624–1.643)	0.959
Mechanical Ventilation in the first 24 h (Reference: Present)	2.78 (1.698–4.552)	<0.001
HFNC(Reference: Present)	0.949 (0.52–1.73)	0.864
Plasmapheresis (Reference: Present)	1.099 (0.241–5.003)	0.903
Inotrop (Reference: Present)	1.899 (1.155–3.123)	0.012
ECMO (Reference: Present)	2.208 (0.227–21.49)	0.495
RRT (Reference: Present)	1.882 (0.679–5.218)	0.224
Mode of Nutrition (Reference: Oral)		
Ng/Og	0.294 (0.171–0.506)	<0.001
Gastrostomy	5.08 (2.486–10.379)	<0.001
Development of Feeding Complication (Reference: Yes)	1.497 (0.871–2.574)	0.145
Nosocomial Infection (Reference: Yes)	2.833 (1.624–4.942)	<0.001
VIS	0.983 (0.975–0.992)	<0.001
PIM2 score	0.984 (0.973–0.995)	0.004
PRISM3 score	0.97 (0.947–0.993)	0.010
PRISM3 pdr	0.99 (0.98–1)	0.059
Patient's Age	0.996 (0.993–1)	0.029
Day of Mortality	1.012 (0.977–1.048)	0.505
Length of Stay in the PICU (day)	0.975 (0.965–0.985)	<0.001
Length of Hospital Stay	0.978 (0.969–0.987)	<0.001
Duration of Mechanical Ventilation	0.974 (0.961–0.986)	<0.001

OR, odds ratio; CI, confidence Interval; PICU, pediatric intensive care unit; HFNC, high flow nasal cannula oxygen; NG, nasogastric; OG, orogastric; ECMO, extracorporeal membrane oxygen; RRT, renal replasman treatment; VIS, vasoactive inotrope score; PIM, pediatric index of mortality; PRISM, pediatric risk of mortality; PDR, predicted death rate.

**Table 7:** Factors affecting reaching the target calorie intake at 7 days of admission.

	Univariate	
	OR (95% CI)	*p*
Gender (Reference: Female)	0.918 (0.539–1.56)	0.751
Source of Admission to the PICU (Reference: Direct Admission from Home)		
Admission from the Pediatric Emergency Room	1.662 (0.311–8.885)	0.553
Other Clinics in the Hospital	1.848 (0.335–10.178)	0.481
Transfer from Other Intensive Care Units	2.457 (0.397–15.227)	0.334
Comorbid disease (Reference: Present)	1.87 (0.992–3.524)	0.053
Mechanical Ventilation in the first 24 h (Reference: Present)	4.598 (2.373–8.909)	<0.001
HFNC(Reference: Present)	0.912 (0.478–1.74)	0.779
Plasmapheresis (Reference: Present)	3.919 (1.16–13.237)	0.028
Inotrop (Reference: Present)	2.057 (1.197–3.536)	0.009
ECMO (Reference: Present)	1.49 (0.153–14.546)	0.731
RRT (Reference: Present)	1.767 (0.608–5.132)	0.295
Mode of Nutrition (Reference: Oral)		
Ng/Og	0.218 (0.103–0.46)	<0.001
Gastrostomy	0.102 (0.036–0.291)	<0.001
Number of Days without Feeding (Reference: Present)	2.525 (1.237–5.152)	0.011
Development of Feeding Complication (Reference: Yes)	2.324 (1.347–4.01)	0.002
Mortality (Reference: Present)	3.829 (1.72–8.526)	0.001
Nosocomial Infection (Reference: Yes)	5.115 (2.915–8.974)	<0.001
VIS	0.982 (0.972–0.993)	0.001
PIM2 score	0.986 (0.974–0.998)	0.019
PRISM3 score	0.971 (0.946–0.997)	0.026
PRISM3 pdr	0.987 (0.977–0.997)	0.011
Patient's Age	0.998 (0.994–1.002)	0.295
Day of Mortality	0.982 (0.951–1.015)	0.289
Length of Stay in the PICU (day)	0.971 (0.959–0.982)	<0.001
Length of Hospital Stay	0.972 (0.961–0.982)	<0.001
Duration of Mechanical Ventilation	0.965 (0.95–0.98)	<0.001

OR, odds ratio; CI, confidence interval; PICU, pediatric intensive care unit; HFNC, high flow nasal cannula oxygen; NG, nasogastric; OG, orogastric; ECMO, extracorporeal membrane oxygen; RRT, renal replasman treatment; VIS, vasoactive inotrope score; PIM, pediatric index of mortality; PRISM, pediatric risk of mortality; PDR, predicted death rate.

Mortality occurred in 7.2% (*n* = 44) of the 614 patients included in the study and the mean time to mortality was 19.02 ± 21.90 days. The factors affecting mortality were examined in univariate and multivariate models using binary logistic regression analysis. In univariate model, the risk of mortality decreased with increasing MUAC Z-score on discharge (OR = 0.871; *p* = 0.027). In univariate model, the risk of mortality decreased with increasing triceps skin fold thickness Z-score on discharge (OR = 0.894; *p* = 0.024). In univariate model, the risk of mortality was 2.723 times higher in patients who did not achieve the target calorie intake at 48 h than those who achieved the target calorie intake (*p* = 0.006). In univariate model, the risk of mortality was 3.829 times higher in patients who did not achieve the target calorie intake at day 7 than those who achieved the target calorie intake (*p* = 0.006). This rate was 8.036 in multivariate model (*p* = 0.043). In univariate model, the risk of mortality was 2.004 times higher in patients with malnutrition according to the weight-for-age than those who had normal nutritional status (*p* = 0.030). The risk of mortality was lower in patients who received early enteral nutrition (OR = 0.321; *p* = 0.001) (Table 8).

**Table 8:** Factors affecting mortality.

	Univariate		Multivariate	
	OR (95% CI)	*p*	OR (95% CI)	*p*
Body Weight Z-score on Discharge	0.883 (0.78–1)	0.050	1 (0.308–3.251)	0.999
MUAC Z-score on Discharge	0.871 (0.77–0.985)	0.027	0.533 (0.27–1.053)	0.070
Triceps Z-score on Discharge	0.894 (0.81–0.985)	0.024	0.68 (0.391–1.184)	0.173
Target Calorie 48h (Reference: Yes)	2.723 (1.339–5.536)	0.006	0.41 (0.016–10.613)	0.591
Target Calorie 7 Days (Reference: Yes)	3.829 (1.72–8.526)	0.001	8.036 (1.063–60.741)	0.043
Body Weight Z-score on Admission to PICU	0.903 (0.798–1.022)	0.106	0.905 (0.171–4.786)	0.907
Height Z-score	0.897 (0.787–1.024)	0.107	0.956 (0.225–4.055)	0.951
Head Circumference Z-score	0.893 (0.708–1.128)	0.343	1.269 (0.865–1.863)	0.223
Body Mass Index Z-score	0.998 (0.961–1.036)	0.906	1.406 (0.426–4.641)	0.576
Weight-for-Height (Reference: Normal)	1.184 (0.638–2.199)	0.592	0.576 (0.038–8.728)	0.690
Nutritional Status According to the BMI Z-score (Reference: Normal)	1.405 (0.76–2.596)	0.278	2.698 (0.231–31.511)	0.429
Weight-for-Age (Reference: Normal)	2.004 (1.069–3.759)	0.030	1.533 (0.061–38.245)	0.795
Height-for-Age (Reference: Normal)	1.748 (0.944–3.239)	0.076	0.835 (0.067–10.437)	0.889
Duration of Malnutrition (Reference: Normal)	1.564 (0.828–2.954)	0.168	0.926 (0.035–24.579)	0.963
According to the Mid-Upper Arm Circumference (Reference: Normal)	1.23 (0.59–2.565)	0.581	4.456 (0.239–83.165)	0.317
According to the Mid-Upper Arm Circumference Percentile (Reference: Normal)	1.128 (0.61–2.084)	0.701	5.118 (0.117–223.447)	0.397
According to the Triceps Skinfold Thickness Percentile (Reference: Normal)	1.225 (0.647–2.321)	0.534	13.098 (0.796–215.551)	0.072
MUAC Z-score	0.906 (0.801–1.026)	0.119	1.889 (0.829–4.307)	0.130
Triceps Skinfold Z-score	0.91 (0.824–1.005)	0.063	1.594 (0.804–3.159)	0.182
According to Early Enteral Feeding Status (Reference: Absent)	0.321 (0.159–0.646)	0.001	2.378 (0.245–23.119)	0.455

MUAC, mid upper arm circumference; PICU, pediatric intensive care unit.

Nosocomial infections occurred in 13.7%of the patients (*n* = 84). The most common infections were bloodstream infections occurring in 9.4% (*n* = 58) followed by ventilator-related pneumonia and urinary tract infections occurring in 6.2% (*n* = 38) and 3.7% (*n* = 23) of the patients, respectively.

The mean length of stay in the intensive care unit was 11.14 ± 15.92 days, the mean length of hospital stay was 18.58 ± 19.07 days, and the mean duration of mechanical ventilation was 11.11 ± 17.77 days. The independent variables affecting the duration of stay in the PICU were examined using linear regression analysis. The length of stay in the PICU increased by 0.062 with increasing triceps skin fold thickness Z-score (*p* = 0.044). The length of stay in the PICU was 0.891 times higher in patients who did not achieve the target calorie intake at day seven (*p* < 0.001).

## Discussion

The present study found that the risk of mortality was lower, the length of stay in the intensive care unit and the hospital was shorter, and the duration of mechanical ventilation was shorter in patients who started on early enteral nutrition, who achieved the target calorie intake at the end of 48 h and 7 days, who did not have malnutrition and those who had high triceps skinfold thickness Z-score. The present study is the first prospective, multicenter study involving pediatric intensive care units in Turkey evaluating nutritional status in critically ill patients and the effect of nutritional status on clinical outcomes. There is an increased risk of malnutrition in critically ill patients who have limited calorie intake throughout their stay in the hospital. In our study patients, malnutrition before or upon admission to the intensive care unit may have developed in relation to various factors such as impairment in nutritional status, inability to take necessary nutrients and vomiting that develop secondary to medical condition. Malnutrition can also develop due to inability to feed during the hospital stay, loss of nutrients, delays in or the inability to deliver necessary nutritional therapy. In order to maintain optimal nutritional status during a critical illness, the initiation of appropriate nutritional therapies involving the administration of energy, proteins, lipids, micronutrients and vitamins at optimal amounts and through an appropriate route is required (3).

In a multicenter study conducted in Brazil, a total of 363 critically ill patients were evaluated. In their study, 62.3% of the patients were male; the mean age was 11.3 months; the reason for admission was medical causes in 75.2%; respiratory conditions were the most common diagnoses occurring in 42.2%; 45.7% had an underlying chronic condition, 76.4% initiated on feeding in the first 48 h; and the mortality rate was 5.5%(16). In the present study, 54.7% of the 614 patients were male with a mean in the entire study group of 80.17 months; the reason for admission was medical causes in 76.2%; 23.3% of the patients were admitted due to respiratory failure; and 62.7% of the patients had an underlying chronic condition. Of the present patients, 72.3% initiated on feeding in the first 48 h and the mortality rate was 7.2%. The rate of underlying chronic conditions was higher among the intensive care unit patients in Turkey, while the other parameters were similar to those reported by the study conducted in Brazil (16).

The rate of malnutrition is critically ill patients has been reported to be ranging between 40% and 70% (17–21). In a study conducted in four intensive care units in Adana province of Turkey, the rate of malnutrition was reported to be 41%, while no significant difference was reported between anthropometric measurements upon admission and on discharge from the intensive care unit (22). The rate of malnutrition classification which weight for age in the present study according to the data collected from the entire country was 45.4% (*n* = 279), and no statistically significant difference was noted between anthropometric measurements obtained upon admission to and on discharge from the intensive care unit. In a study of 951 pediatric patients involving females at a rate of 51.6%, the rate of acute malnutrition was 21.3% and the rate of chronic malnutrition was 41.3% (23). In a study evaluating the data of a total of 293 pediatric patients in the neonatal and pediatric intensive care unit in the Netherlands, the rate of malnutrition was found to be 24%, and when the patients were evaluated according to the duration of malnutrition, 15% had acute, 20% had chronic and 24% had acute/chronic malnutrition. Of the reported patients, 32% were critically ill patients above the age of one month (3). When pediatric patients in Turkey were classified according to the duration of malnutrition, 15% had acute, 23% had chronic and 15.6% had acute/chronic malnutrition.

Although the term malnutrition is used to denote individuals with undernutrition in our daily practice, the term is not specific to undernutrition but also encompasses overweight and obese patients. It was reported that 13% of the patients admitted to the pediatric intensive care units were either overweight or obese according to the BMI Z-score (24, 25). This rate was 6.9% in a study conducted in Brazil (16) and 6.8% in the present study. In retrospective review of 243 pediatric intensive care unit patients in Ethiopia, this rate was reportedly around 11% (26). In a multicenter cohort study of 1,622 patients undergoing mechanical ventilation in 16 countries, nutritional status based on the BMI data was found to be related to clinical outcomes such as the number of ventilator-free days, nosocomial infections and mortality (1). In the same study, 27.9% of the patients were reported to overweight or obese (1). In the present study that used mid-upper arm circumference and triceps skin fold thickness Z-scores to evaluate nutritional status in critically ill patients, the risk of mortality decreased with increasing Z-scores. In another study, mortality remained unaffected with increasing severity of malnutrition, whereas the severity of malnutrition was found to be related to the length of stay in the intensive care unit and prolonged duration of mechanical ventilation (17).

Achieving optimal calorie and protein intake in critically ill patients by early initiating optimal enteral feeding is associated with an improvement in clinical outcomes (27). It is recommended that enteral nutrition be initiated within 24–48 h after admission to the intensive care unit in hemodynamically stable children with a functional gastrointestinal tract if there is no contraindication such as vomiting, abdominal distension and gastrointestinal hemorrhage, and this approach is referred to as early enteral nutrition (28). In our country, 40.6% of critically ill patients initiated on enteral nutrition in the first 24 h after admission and this rate was 72.3% at the end of 48 h. At the end of one week, 91.5% of our patients started on enteral nutrition. The target calorie intake at the end of 48 h was achieved in 86.8% of our patients, whereas 81.6% of the patients continuing their stay in the intensive care unit at the end of one week achieved the target calorie intake. The analysis of diagnoses among critically ill patients who did not achieve the target calorie intake at both time points revealed that trauma and respiratory failure were the most common diagnoses. The rate of complications associated with enteral feeding was 19.9% in our patients, the most complications being vomiting followed by abdominal distension, electrolyte imbalance, diarrhea, gastrointestinal hemorrhage and constipation. It was observed that patients receiving mechanical ventilation support, inotropic therapy and those with high mortality scores were less likely to achieve the target calorie intake. Intubation/extubation procedures and intolerance to enteral nutrition appeared to be the most common reasons when the factors associated with a delay in enteral nutrition were evaluated. In a multicenter, cross-sectional study, mechanical ventilation, disease severity, interventional procedures and gastrointestinal diseases were identified as clinical risk factors associated with a delay in enteral nutrition (29). Other studies have reported that enteral nutrition had been interrupted in 30%–42% of pediatric intensive care unit patients due to intolerance to enteral nutrition, extubation and intubation procedures, bedside interventions, and radiological and surgical procedures (30, 31).

It was found in the present study that 6.5% of the patients started on parenteral feeding within the first 24 h after admission to the intensive care unit and this rate was 8% at the end of one week. However, available guidelines on nutrition do not recommend initiation of parenteral feeding within 24 h after admission to the intensive care unit (5, 28). In the PEPaNIC study, initiation of parenteral feeding within 24 h after admission to the intensive care unit was defined as early parenteral nutrition and initiation of parenteral feeding after seven days was defined as late parenteral nutrition, and the outcomes of early and later parenteral nutrition were compared in critically ill patients. The rate of infections, duration of mechanical ventilation and the length of stay in the intensive care unit were significantly lower in the late parenteral nutrition cohort (32, 33). In a study evaluating nutritional status in 95 critically ill patients, the rate of parenteral nutrition was 7.7% and early parenteral nutrition was initiated in 33% of the patients in a mean duration of four days (1–6 days) (34).

There is an increased risk of malnutrition in critically ill patients who have limited calorie intake throughout their stay in the hospital. It is recommended that critically ill patients should receive a minimum of 54–58 kcal/kg/day nutritional support in order to avoid a catabolic state and maintain protein and energy support as required (28). In the present study, the patients had a mean calorie intake of 57.39 ± 32.97 kcal/kg/day as recommended by the guidelines. Protein intake must be at least 1.5 gr/kg/day in pediatric intensive care unit patients to avoid negative protein balance (5, 28). Our patients received 1.6 gr/kg/day protein support. A study investigated the nitrogen and energy requirement using indirect calorimeters and by performing more than 400 measurements in patients undergoing mechanical ventilation. At the end of this study involving 74 critically ill patients, the authors concluded that minimum protein intake must be 1.5 gr/kg/day and minimum calorie intake must be 58 kcal/kg/day (35).

The present study found that the risk of mortality was lower, the length of stay in the intensive care unit and the hospital was shorter, and the duration of mechanical ventilation was shorter in patients who started on early enteral nutrition, who achieved the target calorie intake at the end of 48 h and 7 days, who did not have malnutrition and those who had high triceps skinfold thickness Z-score. In a study involving 59 pediatric intensive care units from 15 different countries and evaluating optimal protein intake and clinical outcomes in 1,245 critically ill patients undergoing mechanical ventilation, the delivery of daily protein support as required was associated with decreased risk of mortality (36). In another study examining 385 pediatric intensive care unit patients, the rate of malnutrition was 45.5%, and malnutrition was found to be associated with prolonged mechanical ventilation but no association with mortality and the length of stay in the intensive care unit was reported (37). In a multicenter, retrospective study, reaching 25% of the target calorie intake at the end of the first 48 h after admission to the intensive care unit and the initiation of early enteral nutrition were found to be associated with an increased probability of survival compared to late enteral feeders (38). In a study conducted in Turkey evaluating the initiation of early enteral nutrition and achievement of the target calorie intake in the early period, the data of 95 critically ill patients from nine pediatric intensive care units was examined. Early initiated feeding (EIF) was defined as the initiation of enteral nutrition within 24 h after admission to the intensive care unit, and early reached target enteral nutrition (ERTEN) was defined as the receipt of 25% of the total energy requirement at the end of the first 48 h. In the scope of the above-mentioned study, 42% of the patients had respiratory failure and the mortality rate was 16.8%. When the patients were evaluated for nutritional status, 47.4% were in the EIF group; the rate of enteral feeding at day two was 72.6% and 45.3% of the patients were in the ERTEN group. The authors reported that early achieving the target calorie intake decreased mortality (39).

The present study has some limitations. The sample size was small because not all pediatric intensive care units in Turkey participated in the study and there was a small number of patients included in the study. Calorie and protein intake of the patients was evaluated but the effects of other micronutrients, lipids and other elements of nutrition were not studied. In addition, there may be differences in the feeding protocols and practices of the clinics.

## Conclusion

The present study found that the risk of mortality was lower, the length of stay in the intensive care unit and the hospital was shorter, and the duration of mechanical ventilation was shorter in patients who started on early enteral nutrition, who achieved the target calorie intake at the end of 48 h and at 7 days, who did not have malnutrition and those who had high triceps skinfold thickness Z-score. Due to the fact that inadequate and imbalanced nutrition can prolong disease course and increase the length of stay in the intensive care unit and the hospital, timely and appropriate nutritional support may reduce morbidity and mortality in critically ill children. Critically ill patients followed up in the intensive care units require evaluation of the nutrition status, early detection of malnutrition and close monitorization of the patients for the need of nutritional interventions by calculating the energy and protein requirements. The present study is the first in Turkey to evaluate nutritional status, nutritional follow-up and the effects of nutritional therapies on prognosis in pediatric intensive care unit patients, and the data deriving from the present study would be of great importance in the planning of future studies, patient follow-ups and arrangement of treatment protocols.

## Data Availability

The original contributions presented in the study are included in the article, further inquiries can be directed to the corresponding author.
